# Self-assembly behaviours of peptide–drug conjugates: influence of multiple factors on aggregate morphology and potential self-assembly mechanism

**DOI:** 10.1098/rsos.172040

**Published:** 2018-04-11

**Authors:** Qin Fan, Yujie Ji, Jingjing Wang, Li Wu, Weidong Li, Rui Chen, Zhipeng Chen

**Affiliations:** College of Pharmacy, Nanjing University of Chinese Medicine, Nanjing 210023, People's Republic of China

**Keywords:** peptide–drug conjugates, self-assembly, nanoribbon

## Abstract

Peptide–drug conjugates (PDCs) as self-assembly prodrugs have the unique and specific features to build one-component nanomedicines. Supramolecular structure based on PDCs could form various morphologies ranging from nanotube, nanofibre, nanobelt to hydrogel. However, the assembly process of PDCs is too complex to predict or control. Herein, we investigated the effects of extrinsic factors on assembly morphology and the possible formation of nanostructures based on PDCs. To this end, we designed a PDC consisting of hydrophobic drug (*S*)-ketoprofen (Ket) and valine–glutamic acid dimeric repeats peptide (L-VEVE) to study their assembly behaviour. Our results showed that the critical assembly concentration of Ket-L-VEVE was 0.32 mM in water to form various nanostructures which experienced from micelle, nanorod, nanofibre to nanoribbon. The morphology was influenced by multiple factors including molecular design, assembly time, pH and hydrogen bond inhibitor. On the basis of experimental results, we speculated the possible assembly mechanism of Ket-L-VEVE. The π–π stacking interaction between Ket molecules could serve as an anchor, and hydrogen bonded-induced β-sheets and hydrophilic/hydrophobic balance between L-VEVE peptide play structure-directing role in forming filament-like or nanoribbon morphology. This work provides a new sight to rationally design and precisely control the nanostructure of PDCs based on aromatic fragment.

## 1. Introduction

Peptide–drug conjugates (PDCs) use drugs as molecular building blocks to construct self-delivering supramolecular nanomedicine, which have received much attention over the last two decades [1–3]. The drug loading content of PDCs is considerably higher than other drug delivery systems [4–7]. As reported by Cui and co-workers, a well-defined supramolecular filament formed by paclitaxel drug amphiphile has been demonstrated to contain a fixed 41% paclitaxel loading [8]. Besides, PDCs could avoid premature degradation and rapid clearance without compromising the therapeutic efficacy of parent drugs due to the feature of self-assembly. For instance, a typical camptothecin-based PDC has been proven to develop into high drug-loading nanostructures, preventing the rapid generation of the free drug [9]. In another work, a peptide–taxol conjugate released drug slowly for up to 1 month which significantly inhibited tumour growth [10]. Therefore, PDCs show enormous potential of systemic cancer treatment, *in situ* anti-inflammatory treatment and diagnostic therapy.

In order to design more rational self-assembly nanostructure, the assembly mechanism of PDCs is necessary to be considered [9–13]. As a large number of peptide-based assembly materials have been reported, many attempts have been made to explain their spontaneously aggregation behaviour and assembly mechanism [14–16]. An experiment on 16-residue peptide (RADA-16-I) was carried out by the Zhang group, and the result indicated the molecules aggregation was driven by hydrogen bond between peptide-stretched backbone, consequently forming short nanofibrils [17]. Hu *et al*. [18] have revealed that electrostatic interaction between the terminal charges of peptides could affect the twist degree of nanofibres or nanobelts. However, the assembly of PDCs is more complex than pure peptide in terms of structure and mechanism, and few reports paid attention to the assembly mechanism of PDCs. The properties of drugs are significantly different from those of amino acids or aliphatic chains. This means that the morphologies constructed by PDCs tend to be more difficult to predict. Thus, developing deep understanding of PDCs' mechanisms is necessary. This study aims to be a guide for designing new rational structures and regulating the morphology precisely.

In the previous work, we reported a hydrogel-forming Ket–peptide conjugate that has the capability to treat arthritic diseases [19]. Ket is one of the non-steroidal anti-inflammatory drugs, and its peptide conjugates enhanced selectivity to COX-2 and reduced the side effect in comparison with Ket itself. Here, we focus on the assembly mechanism of Ket–peptide conjugates which may explain its better effect compared to Ket. Several parameters, such as pH, assembly time, concentration and hydrogen bonds inhibitor, were used to analyse the assembly behaviour and consequently, the possible mechanism was developed. Our work can help to understand the key factor for PDCs design, offering guidance on designing functional nanomedicine with suitable morphologies.

## 2. Experimental details

### 2.1. Materials

Oligomeric peptide with valine–glutamic acid dimeric repeats (L-VEVE) and (*S*)-ketoprofen-based PDCs (Ket-L-VEVE) were synthesized by Nanjing Aite Biotechnology Co. Ltd with purity above 95%. Pyrene was acquired from Suzhou Industrial Park Bomeida Reagent Instrument Co. Ltd. HPLC-grade methanol was purchased from E. Merck (Merck, Darmstadt, Germany). Other chemical reagents were obtained from Nanjing Shoude Equipment Co. Ltd. All experiments were performed using 18 MΩ H_2_O purified by Milli-Q system.

### 2.2. Determination of molecular dissociation constant

The pKa of Ket-L-VEVE was calculated by the Titrator module and Dispenser module of Sirius (Sirius Analytical Ltd). According to the structure of Ket-L-VEVE, the carboxyl groups on the glutamic acid were not closed to the chromophore of ketoprofen. So, potentiometric titration was chosen to test samples by adding a small amount of co-solvent (MeOH), then using the Yasuda–Shedlovsky extrapolation formula to gain pKa values.

### 2.3. Zeta potential

Zeta potential was measured by Zetasizer Nano ZS90 (Malvern Instruments Ltd, UK). About 1 M HCl and 1 M NaOH were added into PDCs solution to make different pH (pH 1, 3, 5, 7, 9, 11). Then the surface charge variation of PDCs was tested. All groups were assayed in triplicate.

### 2.4. Determination of critical aggregation concentration

Florescence method using pyrene as a probe was performed to monitor the formation of nanostructure in order to determine the critical aggregation concentration (CAC). A volume of 50 µl of pyrene solution (6 × 10^−4 ^M) in methanol was, respectively, added into nine centrifuge tubes. Then methanol in tubes was evaporated overnight in dark condition. Different concentration of PDCs solutions ranging from 3.5 × 10^−6^ to 2.1 mM were mixed with pyrene, and the concentration of pyrene was finally fixed at 6 × 10^−6^ M. After stirring for 24 h at room temperature, a fluorescence spectrophotometer (Shimadzu RF-5301PC) was used to measure the fluorescence intensities of pyrene. At an excitation wavelength of 334 nm, the samples were scanned with an emission wavelength from 350 to 500 nm. Fluorescence intensity ratios of pyrene at *I*_383_/*I*_373_ (*I*_1_/*I*_3_) were plotted against the log of the concentration of each sample.

### 2.5. Transmission electron microscopy

In order to investigate the characteristic of self-assembled morphology, samples were observed by transmission electron microscopy (TEM, Hitachi, Japan). In various experiments, all concentrations of PDCs were 500 µM except specially mentioned. To prepare TEM samples, 7 µl of the appropriate solution was placed onto a copper grid (400 meshes) coated with a carbon membrane, and the excess solution was wicked away with filter paper. Then, uranyl acetate aqueous solution 2.0% (w/v) was deposited on the surface for 1 min. The excess solution was blotted up through a filter slightly. The grids were dried under ambient environment for at least 3 h before TEM observation.

### 2.6. Circular dichroism

The circular dichroism (CD) spectrum was collected by a Jasco 710 spectropolarimeter to deduce the secondary structures of samples in aqueous solution. The tested concentration of PDCs was 500 µM in water. Pure water solution was used to assess the baseline CD signals and all spectra were corrected by subtracting the baseline.

### 2.7. Fourier transform infrared spectroscopy

The infrared spectrum of the sample was recorded by TENSOR-37 (Brucker, Germany) and the spectrum was scanned from 1500 to 1800 cm^−1^ at 4 cm^−1^ resolution.

## 3. Results and discussion

### 3.1. Influence of molecular structure

**Figure 1.**
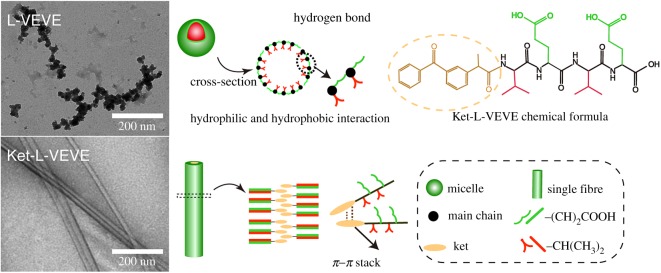
Representative TEM images and graphic illustration of the self-assembly L-VEVE and Ket-L-VEVE in water for 24 h.

In order to investigate the assembly behaviour influenced by molecular structure, the Ket-L-VEVE and pure peptide L-VEVE were assembled under the same conditions by dissolving in water (500 µM, pH 7) overnight at 37°C. As shown in figure 1, long nanofibres or nanoribbons with the width of 50.44 ± 5.50 nm were observed in the PDC solution, while pure peptide tended to form small nanospheres rather than fibres, and the mean diameter was only 20.61 ± 2.36 nm. The evidence from this study indicated that the presence of drug molecular apparently changed the assembly behaviour of peptide. L-VEVE is an oligomeric peptide containing alternating hydrophobic and hydrophilic parts and its isoelectric point was 3.1 calculated by the free peptide calculator. Since pH 7 was quite beyond the isoelectric point, molecular dissociation made glutamic acid (E) ionic, increasing the feature of hydrophilicity. Then, due to hydrophilic–hydrophobic property between the side chains and the hydrogen bond of amino acids, L-VEVE aggregated spontaneously. The intrinsic chirality may lead to a certain helix of the main chain which was easy to form hydrogen bond between the nanospheres. Thus, the small sticky nanospheres were not well dispersed and formed their regular aggregative structure. However, the morphology changed enormously after conjugating Ket with peptide. Ket is an aromatic drug with two benzene rings, which is easy to form π–π stacking [12,20]. We presumed the π–π stacking effect of Ket-driven PDC molecular to aggregation, which was quite different from the assembly behaviour of L-VEVE. Thus, this finding provided further evidence that π–π stacking of aromatic drug governs early stages of the self-assembly process and makes a contribution to stabilize the nanostructure, which was also founded by Kang *et al*. [21].

### 3.2. Influence of concentration

**Figure 2.**
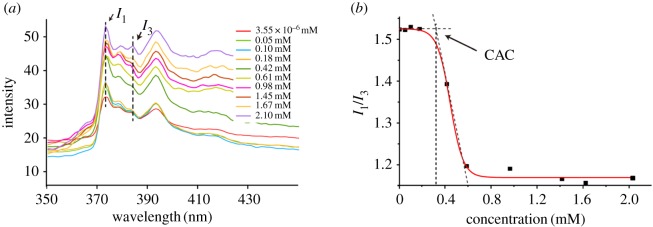
Fluorescent emission spectra of purine (*a*) with the increased concentration of Ket-PDCs and (*b*) the CAC of Ket-PDCs.

During the experiment, we found that PDCs could form nanostructure in high concentration, while no morphology was found in low concentration. Therefore, the CAC of Ket-L-VEVE was determined to ensure the assembly. In this study, fluorescent dye pyrene was used to study the effect of concentration on self-assembly [22,23]. Owing to poor water solubility, pyrene could enter into the hydrophobic core of the assembly structure in which fluorescence can be detected. As the concentration of PDCs solution decreased gradually, the fluorescence intensity became weak, which indicated that the assembly structure reduced (figure 2*a*). And when the PDCs concentration decreased to a certain value, the fluorescence intensity would not change anymore. The intensity of two peaks (*I*_373,_ *I*_383_) was plotted to extrapolate the sharply changed point, and the CAC value of PDCs was 0.32 mM (figure 2*b*). It showed Ket-PDC molecular began to aggregate and assembled at the concentration of 0.32 mM. Moreover, this result also proved that there were hydrophobic moieties in the assembly structure which were mainly attributed to the aromatic part of the drug, as mentioned above.

### 3.3. Influence of assembly time

**Figure 3.**
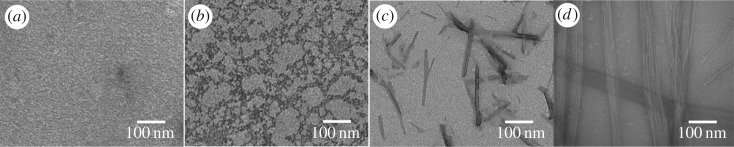
TEM images of Ket-L-VEVE at different assembly time of (*a*) 0 h, (*b*) 1 h, (*c*) 4 h and (*d*) 24 h at a concentration of 500 μM in water.

To investigate the assembly process of PDCs during the time, the morphology of self-assembly Ket-L-VEVE was monitored at different times after dissolving in water at a concentration of 500 µM. Seen from figure 3, no obvious structure was observed after the sample immediately dissolved (figure 3*a*). Then, worm-like morphology was observed after letting them sit about 1 h (figure 3*b*). This massive irregular aggregation structure which well distributed in sight may be the intermediate state which indicated the coming-on assembly. With the time extending to 4 h, the massive undefined structure disappeared, replaced by rod-like fragments with different length. Interestingly, when assembled after 24 h, regular nanoribbons assembled by several nanofibres became the main structure. The disappearance of the rod-like structure short fibres indicated it was a kind of pre-assembly structure and probably reassembled into long fibres via end-to-end assembly, then the fibre–fibre aggregation into bundles, which was proved by Morbidelli and co-workers [24]. And this nanoribbon morphology was the final structure and remained stable during the following days. Therefore, the assembly process of PDC molecular was time-dependent and the assembly structure was quite stable after the assembly was finished.

### 3.4. Influence of pH

**Figure 4.**
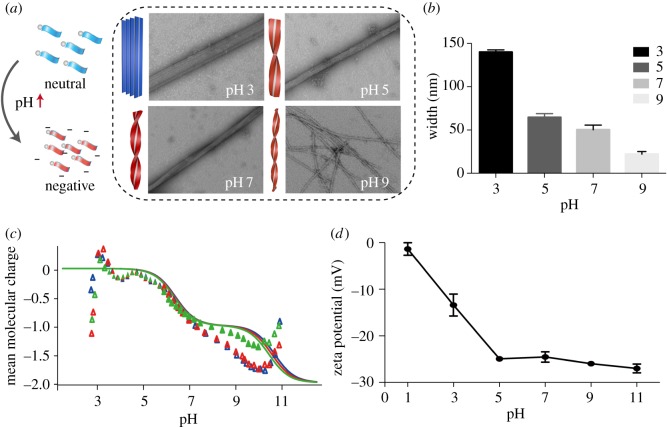
(*a*) TEM image of PDCs assembly for 24 at pH 3, 5, 7 and 9. Scale: 200 nm. (*b*) The statistical width of the nanofibre or nanoribbon in TEM at different pH, (*c*) mean molecular charge caused by carbonate ionization, and (*d*) the zeta potential of assembly PDCs at differentpH.

The pH of the solution is considered to be a crucial factor in supramolecular self-assembly. In this work, the samples were adjusted to different pH value from 1 to 11 to investigate the assembly behaviour. We found major morphology was long fibre or ribbon in pH = 3–9 with subtle change, while no nanostructure existed at pH = 1 or 11. With the pH value increasing from 3 to 9, the wide ribbon became narrow, even to single nanofibre at pH = 9. At the same time, the narrowing ribbon twisted more and more severely (figure 4*a*). The rigid ribbon with no twist was observed in pH 3, and the width was 140.77 ± 2.63 nm. The width of the ribbon decreased gradually from pH = 3 to 9, and came to 22.16 ± 3.05 nm at pH = 9 (figure 4*b*).

Judging from the different morphology influenced by pH, we suspected the assembly behaviour was attributed to different molecular state caused by pH. There are three carboxyl groups in the molecular structure of Ket-L-VEVE, so the carboxyl dissociation state influenced by pH was critical for assembly. The determination of molecular dissociation constant was performed. As the third ionization was very weak, only two dissociation constants could be calculated as pKa_1_ = 3.81 and pKa_2_ = 4.78 (figure 4*c*). Next, the zeta potential of assembly PDCs solution at different pH was examined to explore the surface charge variation and explain the assembly behaviour. From pH 1–11, the zeta potential of PDCs solution changed from neutral to negative (figure 4*d*). This was because glutamic acid residues gradually disintegrated and Ket-L-VEVE transit from molecularity to the ionic state. The zeta potential seemed not to change when pH was above 5, which corresponded to the pKa_2_ value, which showed the molecules were completely dissociative. When pH was closed to the pKa_1_ point, electrostatic repulsion between Ket-L-VEVE molecules was weak, and many molecules tended to easily aggregate into ribbon. As the pH increased, the degree of molecular dissociation increased, and more negative charge occurred. Enhanced electronic repulsion hindered the aggregation of molecules and distorted the entire structure. The lateral growth of nanofibre was limited. Eventually, structure changed from ribbon to single fibres. Therefore, charge repulsion is a useful approach to regulate the aggregation of PDCs and control the final structure.

**Figure 5.**
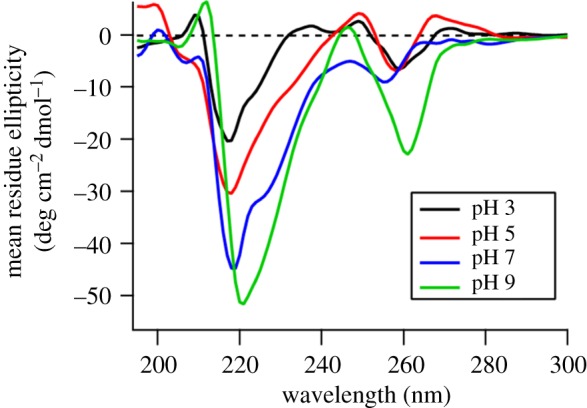
CD spectra of PDCs at pH (black) 3, (red) 5, (blue) 7 and (green) 9 in water.

CD spectroscopy was used to characterize the second structure of PDCs, which could reflect the formations of intermolecular packing and nanostructure twisting. The profile of the CD curve in different pH conditions was almost similar, as shown in figure 5. According to the literature reports, a characteristic negative peak at 216 nm generally indicated a typical β-sheet structure [25]. At pH 3, the CD spectrum had a strong negative band at around 218 nm. As the pH increasing, the maximum negative peak was changed from 218 to 220 nm. These slightly red-shifted behaviours revealed that the twisted β-sheet secondary structure was gradually increasing upon the increase in pH, which was consistent with the results of TEM images that the increasing pH led to form the twisted nanostructure. The observation of two addition peaks between 224 and 300 nm may represent the π–π stacking interaction between aromatic Ket molecules. Moreover, as the pH increased, the increasing charge repulsion tended to change the stable states of PDCs, particularly affecting the β-sheet organization. The intensity change of CD signals on π–π interaction resulted from the formation of the twist structures. Whereas under the formation of nanobelts, the degree of π–π stacking was changed slightly from pH 3 to 7, indicating that π–π interactions regained stability and dominated the supramolecular organization. The π–π interactions may be much stronger in the single fibre morphology at pH 9, resulting in the obvious intensity increase at 224–300 nm.

### 3.5. Interference of hydrogen bond inhibitor

**Figure 6.**
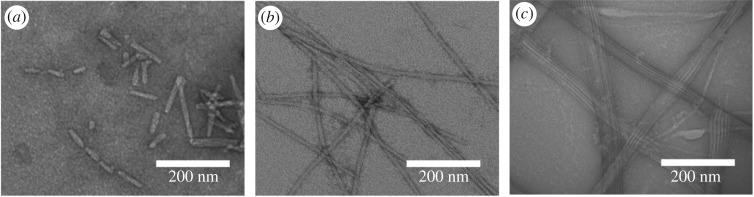
TEM of PDCs assembly after 24 h in the present of 5 M urea (*a*) adding urea at the beginning, (*b*) adding urea after 4 h pre-assemble, (*c*) common situation without urea.

Hydrogen bond between amino acids plays a vital role in peptide assembly process, so it is also thought to take place in the PDCs assembly. Urea, a common hydrogen bond inhibitor, can preferentially break the hydrogen bond between the amino acids, due to strong hydrogen bonds interaction between urea and peptide. In this section, the sample prepared by adding 5 M urea solution showed different morphology after 24 h assembly. If urea was adding at the beginning of the assembly, only several short rods with the length between 23.22 and 75.05 nm could be seen (figure 6*a*). When urea was added after 4 h pre-assembly, long single fibres became the main morphology (figure 6*b*) which indicated the occurred assembly was irreversible and urea could interrupt the following assembly. Without adding urea, the following assembly would go on and finally, structures were much longer and thicker ribbon (figure 6*c*). These results revealed that urea could delay the assembly time from long fibre and affect further growth to ribbon. Therefore, intermolecular hydrogen bond influenced not only the initial stage of the assembly of molecules, but also the advanced stage of the fibre-to-ribbon assembly.

**Figure 7.**
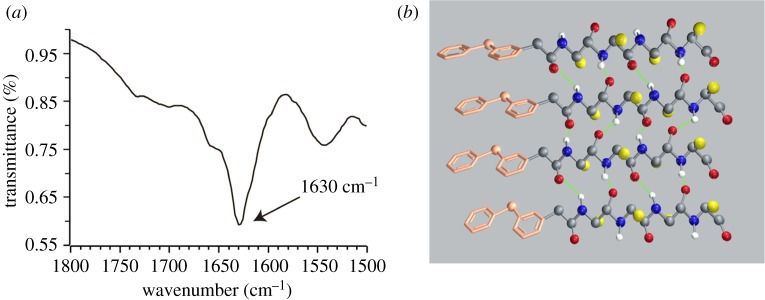
(*a*) FT-IR spectra of Ket-PDC, and (*b*) a model of intermolecular hydrogen bond between Ket-PDCs.

Fourier transform infrared analysis (FT-IR) showed an amide I band near 1630 cm^−1^ (figure 7*a*) indicating the hydrogen bond conformations in β-sheet, which was in agreement with the CD results (figure 5). Our result proved the hypothesis that intermolecular hydrogen bond not only affected the initial assembly of molecules, but also determined fibre–fibre aggregation. Additionally, there could be an increasing twist in β-sheet hydrogen bond which was related to the chirality of amino acids (figure 7*b*).

### 3.6. Assembly mechanism

The aim of the current study was to investigate the assembly-dependent factors and self-assembly mechanism of Ket-L-VEVE. Multiple factors, including molecular design, concentration, time, pH and hydrogen bond inhibitor, have been investigated systematically. We found that Ket-L-VEVE formed short rod at first after dissolving in neutral solution, then as time went on, re-assembled into long nanofibre or nanoribbon. It indicated the nanostructure assembled via an end-to-end and fibre-to-fibre process. The concentration of PDCs determined whether the nanostructures can be formed, and the assembly time determined which nanostructures can be formed. Interestingly, pH-dependent electrostatic interaction could regulate the width of nanofibre owing to the molecular dissociation. Hydrogen bond inhibitor could suppress the assembly process. Therefore, it is a good solution to control the formation of nanostructure via regulating the external factors, providing a new angle for researchers to study the regulation of the PDCs’ self-assembly.

**Figure 8.**
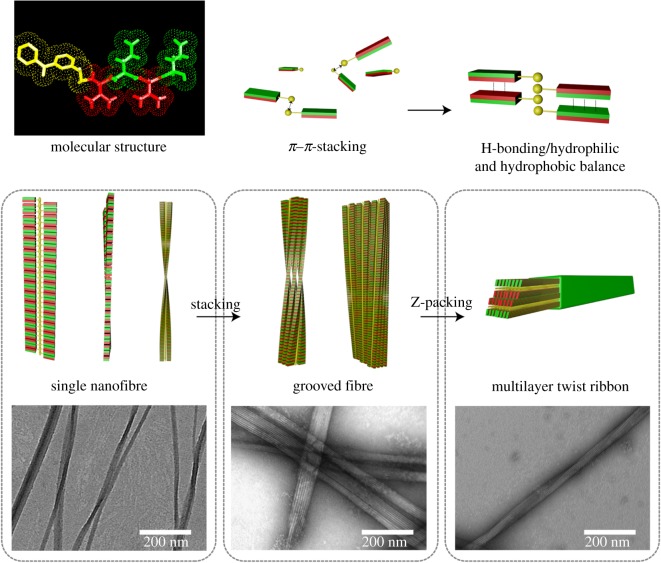
The mechanism of Ket-L-VEVE self-assembly.

Based on the above experimental results, we presented the possible assembly mechanisms of Ket-L-VEVE. Firstly, the π–π stacking originating from Ket molecular served as anchors, which was also the hydrophobic motif and tended to gather in the centre (figure 8). Hydrophobic amino acid (V) and hydrophobic amino acid (E) were alternating permutation as side chains distributed on both sides of the main chain inducing hydrophobic and hydrophilic regions. The hydrogen bonding interaction between peptide chain induced the β-sheet. Under the synergistic action of hydrogen bonding and hydrophilic/hydrophobic equilibrium force, Ket-L-VEVE were rearranged and stacked up in the dimensions and directions of space to form single fibre. As the assembly process went on, hydrogen bonding between fibres resulted in the lateral stacking, and the grooved ribbon was one of the intermediate states formed by the aggregation of single fibres. Finally, multi-layer nanoribbon was formed. The width of the ribbon was closely related to the charge repulsion of molecules. Neutral molecules tended to form flat and rigid ribbon. With the increase in electronic repulsion, the aggregation between fibres was blocked and the pitch of ribbon decreased, leading to the narrower width. Furthermore, due to the intrinsic chirality of amino acids, such nanoribbon had a tendency to twist. With the increase in electrostatic repulsion, the twist trend was more pronounced.

## 4. Conclusion

In this study, our work investigated the self-assembly behaviour of one kind of aromatic drug-based PDCs Ket-L-VEVE and concluded its potential self-assembly mechanism. Multiple factors, including molecular structure, concentration, time, pH and other inhibitor, can give different influence on aggregate morphology of Ket-L-VEVE. The good manipulation of these factors can be used to control the supramolecular assemblies to the wanted nanostructure. This approach can be applied into other PDCs containing aromatic fragment. The principle of aromatic-based PDCs assembly is that aromatic structure is prerequisite to pre-assembly through π–π stacking, and the peptide assist in the reassembly for stable morphology through the synergistic effect of hydrogen bonding and hydrophilic/hydrophobic equilibrium. Our findings provide a manageable strategy for the better investigation of PDCs and a new insight into the mechanism of PDCs assembly. The basic principles can guide the rational design and precisely tailor the subtle structure of PDCs.

## Data accessibility

A complete dataset of measurements is available from Dryad: http://dx.doi.org/10.5061/dryad.ft7c7[26].
